# Surgical alternatives for treatment of developmental dysplasia of the hip in children aged 1–4 years: does open reduction improve the outcomes?

**DOI:** 10.1007/s00590-026-04775-1

**Published:** 2026-05-18

**Authors:** Eugen Cohen, Mariya Fiterman, Bryan Itkowitz, Ali Elaobda, Ron Rotkopf, Muhamad Eldada, Vadim Benkovich

**Affiliations:** 1 Soroka Medical Center, Beer Sheva, Israel; 2 Ben-Gurion University of the Negev, Beer Sheva, Israel; 3 Weizmann Institute of Science, Rehovot, Israel

**Keywords:** Developmental hip dysplasia, Surgical treatment, Outcomes, Complications

## Abstract

**Background:**

Developmental dysplasia of the hip (DDH) remains a therapeutic challenge and treatment strategies remain controversial in children diagnosed after the age of one year.

**Aim:**

The study aim was to evaluate various treatment methods for DDH in children aged 1–4 years and to determine whether open reduction improved outcomes.

**Methods:**

41 children (48 affected hips) aged ≥ 1 year with DDH were included in the study. They were treated surgically between 2012 and 2021 at a tertiary university hospital and had a documented follow up of ≥ 18 months. Patients were divided into two groups: those who underwent closed reduction (CR) and those who underwent open reduction (OR). Femoral shortening, varisation and pelvic extracapsular osteotomies (PO) were performed depending on case and surgeon preference. Outcomes were evaluated radiographically using the Severin classification, functionally using the Children’s Hospital of Oakland Hip Evaluation Score (CHOHES). Avascular necrosis (AVN) if present was documented using the Kalamchi–MacEwen (K&M) classification. Optimal outcomes were defined when: quality of reduction is Severin ≤ 2, CHOHES ≥ 85 and absence of AVN. Statistical analyses were performed using R version 4.3.1, with categorical data compared using the chi-square test.

**Results:**

Optimal outcomes were observed in 19 of 21 hips treated with OR (90.5%) and 19 of 27 hips treated with CR 70.4% (*p* = 0.089). AVN occurred infrequently, and in several cases, resolved during follow-up.

**Conclusions:**

OR with Femoral shortening and PO provided the most favorable outcomes. This approach may minimize AVN, and potentially delay degenerative changes.

## Introduction

Developmental dysplasia of the hip (DDH) is the most common congenital musculoskeletal disorder, as described by Multerer and Döderlein [1]. Hip development progresses rapidly during the first 12 weeks of life, followed by a plateau phase during infancy [2]. Early diagnosis is essential, as treatment initiated in the neonatal period typically yields excellent results. Routine ultrasound screening, as described by Graf, has significantly reduced the need for invasive interventions and lowered healthcare costs [3]. Despite screening programs, cases of late-diagnosed DDH continue to occur due to delayed presentation, failed initial management, or logistical barriers to care. In such cases, the optimal treatment strategy remains controversial [4, 5]. The main objective of this study was to evaluate various treatment methods for DDH in children aged 1–4 years and to determine whether open reduction improved outcomes. The secondary goal was to identify correlations between favorable outcomes and treatment protocols .

## Methods

This retrospective comparative study (Level of Evidence III) was conducted at a tertiary university hospital following institutional ethics committee approval. Children aged 1–4 years who were treated between 2012 and 2021 with a confirmed diagnosis of DDH (ICD-9 code) and had a minimum follow-up of 18 months were included. Patients with syndromic or neurologic causes of hip dysplasia were excluded. Data on demographics, severity of dislocation, treatment strategy, complications, and radiologic and functional outcomes were extracted from the hospital’s digital database.

All patients underwent surgical treatment and were divided into two groups according to the primary procedure: closed reduction (CR) or open reduction (OR). CR was performed under general anesthesia using fluoroscopy and a dynamic arthrography to assess the accuracy of the reduction and the optimal position for cast immobilization. CR was considered satisfactory if the reduction was concentric on arthrography, the medial dye of the contrast medium was less than 7 mm and excessive abduction was not needed to maintain the reduction. The treatment plan included initial CR followed by treatment per surgeon assessment if further intervention was necessary. If satisfactory CR was not achieved, the procedure was converted to OR. In practice, CR was often combined adductor longus tenotomy. In some CR cases, femoral varus osteotomies were performed to increase the stability of the reduction.

OR were performed through a bikini anterior approach, including capsulotomy, ps*o*as release, excision of the ligamentum teres and pulvinar, and transverse ligament incision, followed by capsuloraphy after reduction. Depending on intraoperative findings and surgeon preference, femoral shortening and derotation were performed during the same session. The aim of femoral shortening was to reduce the risk of avascular necrosis (AVN). Derotation osteotomy involves around 30 degrees of external rotation of distal fragment through a subtrochanteric osteotomy to improve the primary stability of the hip by correcting the anteversion. After correction, the osteotomy was fixed by a plate. Pelvic extracapsular osteotomy (PO), Dega or Pemberton, were performed in both OR and CR groups if a shallow acetabulum was clearly identified, namely an acetabular index (AI) >50º. The rationale for PO is as follows: (a) the triradiate cartilage is open in the 1–4 year age group, serves as a hinge to achieve reshaping of the acetabular roof (b) the remodeling capacity in walking age children was considered unpredictable. The decision whether a PO is indicated was made preoperatively. In our institution Dega and Pemberton osteotomies are preferred, as these are incomplete osteotomies, correction is achieved at the level of deformity, their result is an acetabuloplasty, internal fixation is not needed and no operative removal of hardware is required.

The patients were immobilized in a hip spica cast for 6–8 weeks, followed by a cast change under anesthesia and an additional four weeks of immobilization. The severity of dislocation was classified using the Tönnis grading system (Grades I–IV) [6]. Radiographic outcomes were assessed using the Severin classification, where Grades I–II were considered good, Grades III–IVa indicated residual dysplasia, and Grades IVb–VI indicated redislocation [7]. Avascular necrosis (AVN) was identified radiographically and graded according to the Kalamchi–MacEwen classification [8]. Functional outcomes were evaluated using the CHOHES score [9]. The CHOHES is a modification of the Harris Hip Score designed to be applicable in children.The assessment domains are: (a) pain b) function and c) physical examination. The score range is 0-100. An “optimal outcome” was defined when all the following criteria were met: Severin Grade I–II, absence of AVN, and CHOHES score ≥ 85. Statistical analyses were performed using R version 4.3.1, with categorical data compared by the chi-square test. A *p*-value < 0.05 was considered statistically significant.

## Results

**Fig. 1:**
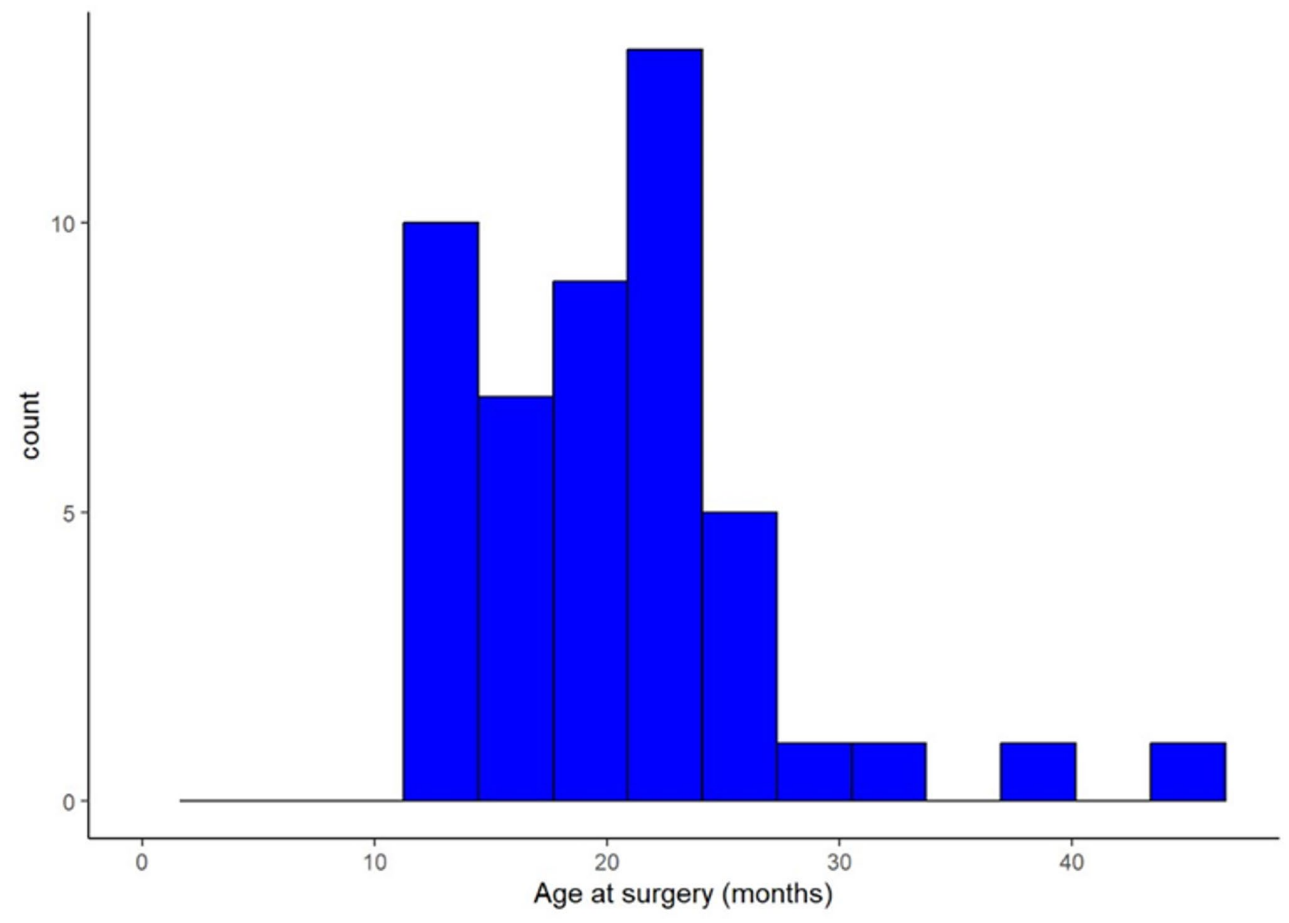
Distribution of age at surgery (months) among children treated for developmental dysplasia of the hip (DDH)

A total of forty-one children (48 hips) met inclusion criteria, with a female-to-male ratio of 38:3. The mean age at surgery was 20.4 ± 6.6 months, and the mean follow-up was 55.1 ± 36.4 months. Of the 48 hips, 27 underwent closed reduction and 21 underwent open reduction (Fig. 1). 19 of 21 hips in the OR group and 14 of 27 hips in the CR group underwent bony procedures: femoral and / or pelvic extracapsular osteotomies (PO).

**Fig. 2:**
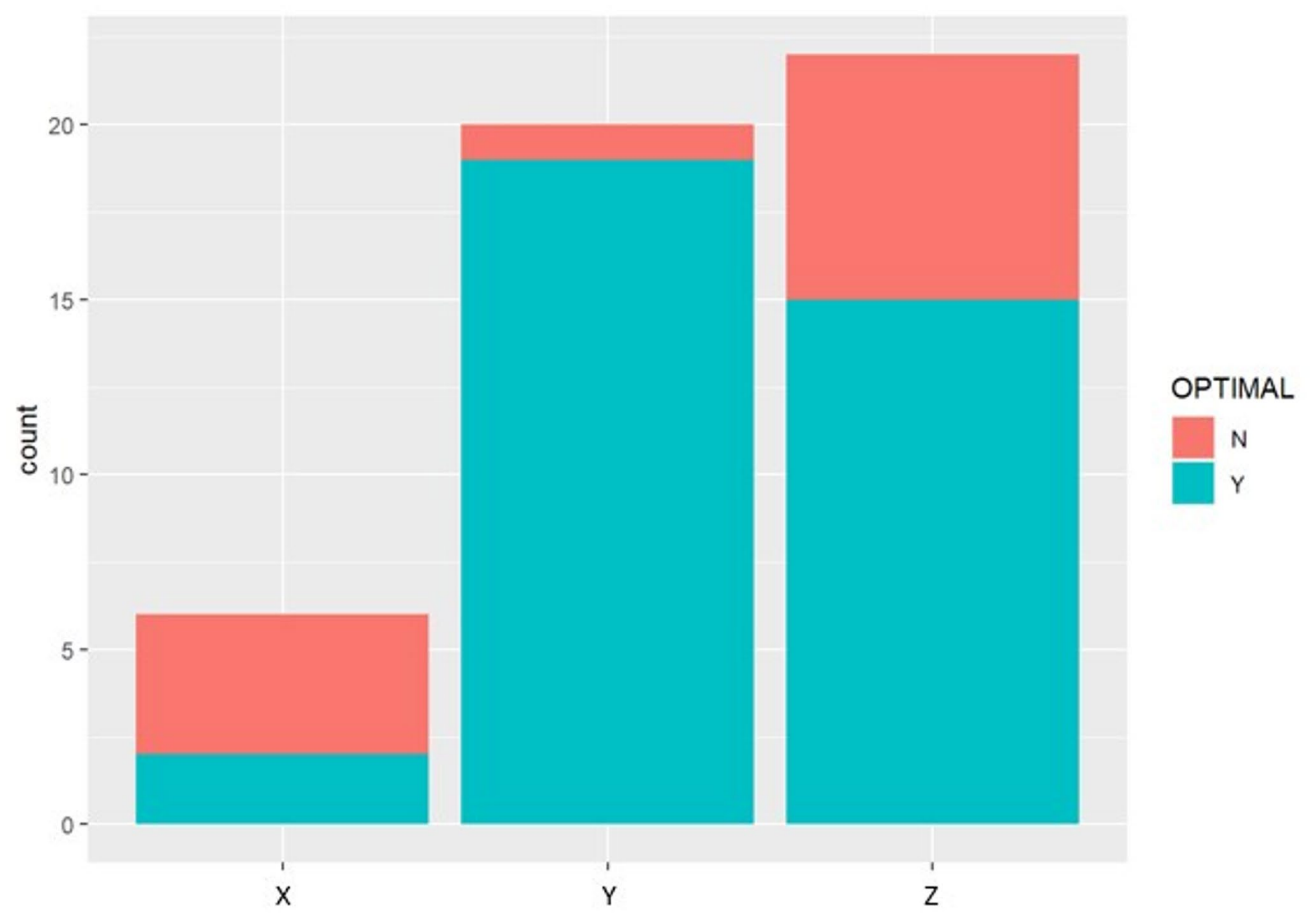
Comparative outcomes of treatment protocols and inter-surgeon variations in results

**Fig. 3:**
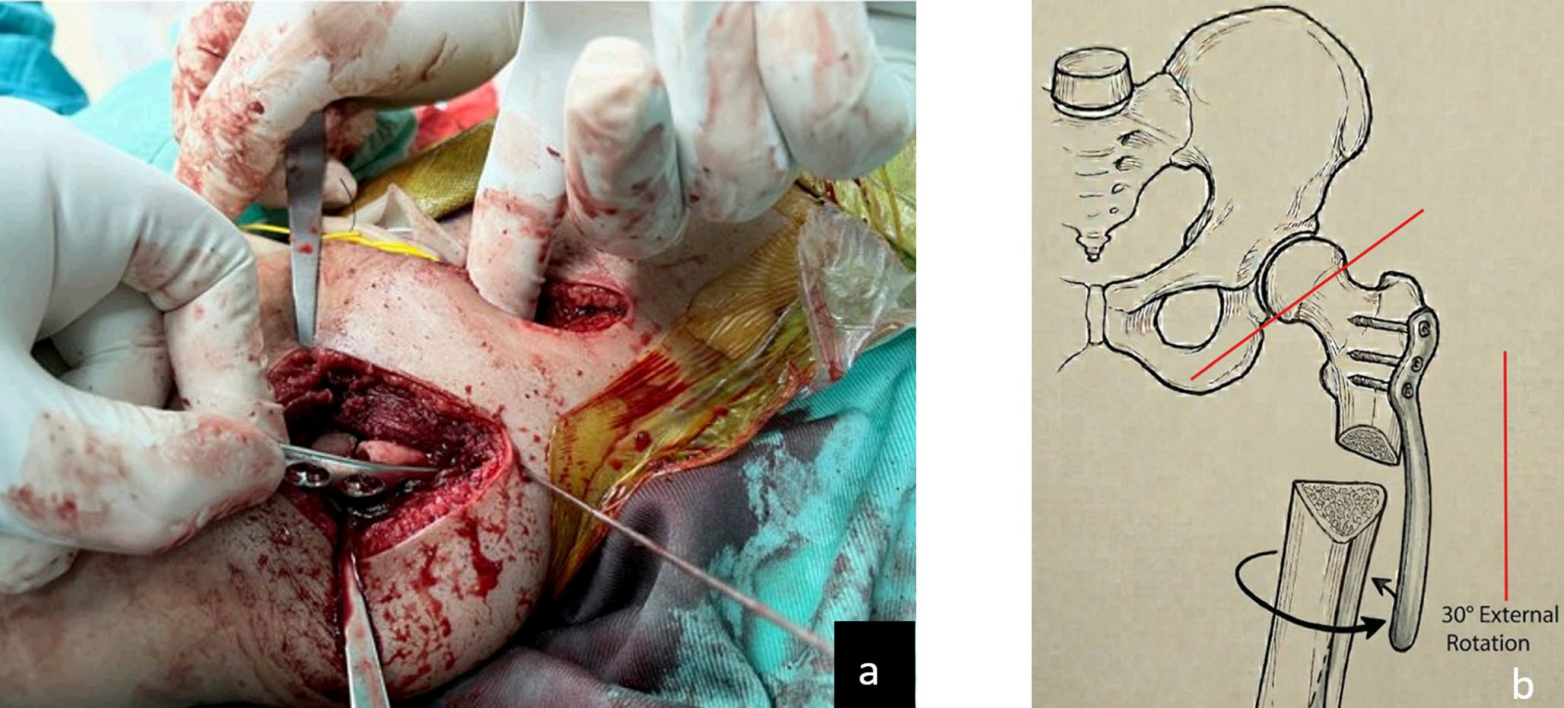
**a**,** b** Key intraoperative step: assessment of hip reduction with one hand while determining the required femoral shortening and derotation with the other hand (**a**). The red lines in the schematic represent the incision lines (**b**)

Optimal outcomes were achieved in 19 of 21 hips (90.5%) following open reduction and in 19 of 27 hips (70.4%) following closed reduction (*p* = 0.089). Redislocation occurred in three hips following closed reduction and one hip following open reduction. A strong but statistically nonsignificant correlation was observed between higher Tönnis grade (III–IV) and suboptimal radiographic outcomes (Severin ≥ IVb, *p* = 0.54) (Table 1). AVN at 18 months was observed in four hips in the CR group and five hips in the OR group. At the final follow-up, several cases demonstrated partial or complete radiographic improvement in AVN signs (Table 2). Three surgeons performed the procedures, each employing a distinct and different protocol. Protocol A (open reduction with femoral shortening, derotation, and PO) yielded optimal results in 19 of 20 hips. Protocol B (closed reduction with varus and extraarticular pelvic osteotomy) achieved optimal results in 15 of 21 hips, while Protocol C (closed reduction with varus only) resulted in optimal outcomes in only two of six hips. These differences were statistically significant (χ² = 4.86, *p* = 0.027) (Fig. 2). Technical review indicated that after capsular release and femoral shortening, the hip should be concentrically reduced and stable between 30–80° of flexion and 30–50° of abduction (Fig. 3). When instability persisted, additional shortening or derotation was performed to achieve intraoperative stability, permitting immobilization in a physiological position.

**Table 1:** Radiographic outcomes according to initial severity (Tönnis and Severin grades)

Initial severity Tonnis grade 1–2 -Nr of cases	Good Radiographic outcomes - Severin grade 1–2	Poor Radiographic outcomes - Severin grade 3–6
	3	0
Initial severity Tonnis Grade 3–4 -Nr of cases	40	5

**Table 2:** Incidence and progression of avascular necrosis (AVN) by treatment type and follow-up period

AVN grade Kalamchi and MacEwen	Follow up 18 M - Closed Reduction	Follow up 18 M - Open Reduction	Last Follow up Closed Reduction	Last Follow up Open Reduction
Grade 1	4	1	2	0
Grade 2	0	1	0	0
Grade 3	0	2	0	1
Grade 4	0	1	0	1
No AVN	23	16	25	19

## Discussion

The implementation of generalized neonatal ultrasound screening has significantly reduced the incidence of late-diagnosed developmental dysplasia of the hip (DDH) in developed countries. For example, in Austria, following the introduction of national screening program, the rate of open reduction decreased to 0.24 per 1,000 newborns while the treatment rate before hip sonography was 13.16% and the invasiveness of treatment dropped [2, 3]. General universal screening by ultrasound is still questioned [2]; it is beyond the objective of the present work to enter the controversy. Despite progress in early diagnose, children presenting at walking age with untreated or residual DDH remain a clinical reality, due to delayed presentation, brace treatment failure, or prolonged waiting times. The optimal treatment strategy for these cases continues to be debated [5, 10–12].

Our study, inspired by the work of Cummings et al. [13], represents one of the largest single-center cohorts of walking-age children with DDH treated in a developed country with an established screening program. Although retrospective, the management philosophy across all surgeons involved was consistent: after one year of age, the potential for spontaneous acetabular remodeling diminishes, and therefore, surgical treatment should aim to achieve anatomic restoration through a single-stage procedure.

The main difference between surgeons was whether open reduction was chosen as the initial approach. The surgeons who performed CR even in walking age children, did so because according current knowledge, the procedure is considered less invasive [13] and does not increase the risk of AVN [5]. More extensive surgery was associated with decreased range of motion [4]. Their decision may also have been influenced by reports of AVN rates as high as 48% after open reduction [14]. Other surgeons, including in our institution, are convinced that OR is required to remove any anatomic impediment and to ensure operative strategy that including OR, pelvic osteotomy (either Pemberton or Salter), and femoral shortening osteotomy can achieve correction of all existing pathological abnormalities in a single operation [11]. Ganger et al., noted that [4] “this approach is considered to be less traumatic for the hip compared with closed reduction and prolonged immobilization”.

**Fig. 4:**
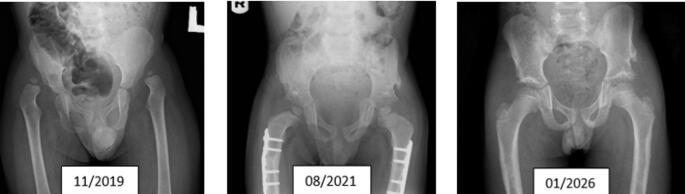
Illustrative case of a late diagnosed patient with DDH. The patient was born in 2017 and diagnosed with bilateral DDH in November 2019. Right hip was operated on in 2020 and left hip in 2021. The radiograph in the middle was obtained 6 months after left hip surgery. The most recent radiograph is from 2026

**Fig. 5:**
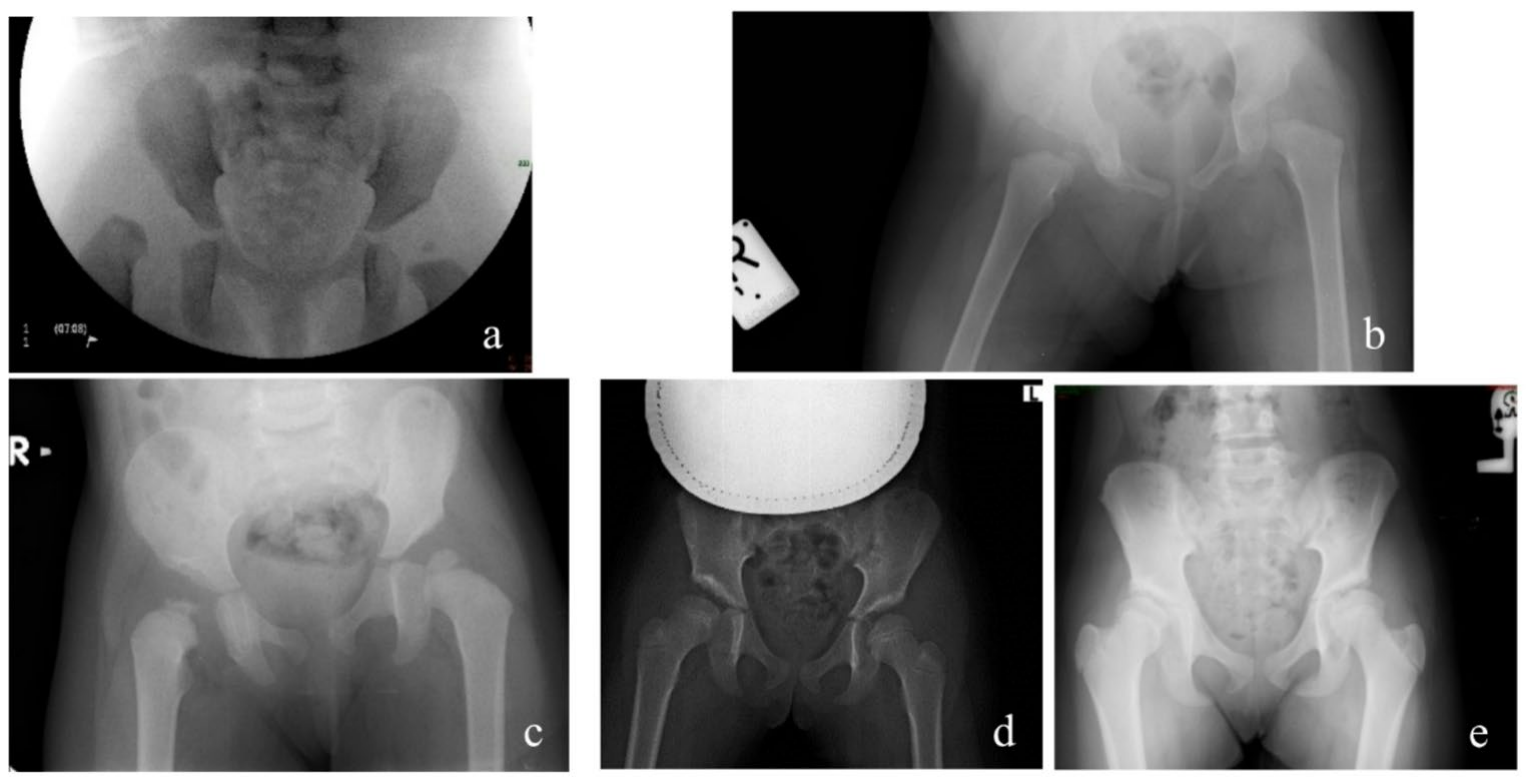
**a–e** Radiographic evidence of spontaneous improvement in avascular necrosis. The child underwent open reduction of the right hip at the age 1.4 years (**a**), At cast removal and 6 months later, signs of AVN can be seen (**b**, **c**). At 5- years follow up AVN had resolved, although the hip remained dysplastic (**d**). Ten years after surgery there is further improvement of dysplasia (**e**)

Consistent with previous studies [10, 11, 15, 16], our results demonstrate that open reduction combined with femoral shortening, derotation osteotomy, and pelvic extracapsular osteotomy (Dega technique) provides the most stable and durable outcomes (Fig. 4). Femoral shortening decreases intra-articular pressure and facilitates reduction, while PO contribute to long-term stability under mechanical load rather than immediate intraoperative stability. We concur with Venkatadass et al. [12] and Forlin et al. [16], who recommend a low threshold for open reduction in children older than 12 months, emphasizing that femoral shortening not only eases reduction but also lowers the risk of avascular necrosis (AVN). Alassaf, compared CR vs. open reduction and pelvic reshaping osteotomy (ORPO) in the age group 18–24 months and found that OR and pelvic osteotomies led to more predictable acetabular remodeling, while about a quarter of CR patients have residual dysplasia [17] .The outcomes of our series were similar. The distinction between proximal femoral growth disturbance and true AVN remains controversial. In this study, AVN was classified using Salter’s criteria as applied by Castañeda et al. [15]. The incidence of AVN (Grades II–IV) did not differ significantly between open and closed reduction groups. Importantly, the rate of AVN in our cohort was lower than that reported by Pospischill et al. [18] and Domzalski and Synder [19]. Our findings align with their observation that femoral shortening has a protective effect against AVN in walking-age children. Similarly, Hussain et al. [20] reported a significant association between older age at presentation and higher AVN risk; our data showed that anatomical restoration (as reflected by Severin I–II) was associated with radiographic improvement or even resolution of AVN changes over time (Fig. 5: radiographs of one of our patients treated by OR). This suggests that restoring congruent joint anatomy can promote remodeling and partial recovery of femoral head vascularity—contrary to Bolland et al. [21], who proposed that childhood AVN is irreversible. Overall, our data support the treatment concept proposed by Forlin et al. [15]: a single-stage procedure including open reduction, femoral shortening, and pelvic extracapsular osteotomy offers the highest likelihood of achieving stable, concentric reduction with minimal morbidity in children aged 1–4 years. Primary intraoperative stability enables immobilization in a physiological position, which may contribute to the low AVN rates and excellent long-term functional outcomes observed in this series.

Limitations of study: (a) This study is limited by its retrospective design and heterogeneity in surgical protocols, follow-up duration (ranging from 18 to 72 months). Each protocol is linked to a specific surgeon and as the study covered 10 years, literature reports appeared during the period and challenged the previous knowledge. Surgeon related factors are a bias, in our opinion possible reflect changes in the approach to late diagnosed DDH during last decade. (b) While acknowledging the inherent limitations of the relatively small sample sizes, we believe the results still demonstrate an interesting trend, even where the differences are not statistically significant. (c) Interobserver variation in radiographic classifications (Severin, Kalamchi-MacEwen, ) may also introduce bias. (d) Although not specifically limited to a particular age group CHOHES was validated [9] or used in children ≥ 8 years [15]. In our cohort CHOHES was used for children as young as 3 years. Assessment of pain may be less precise in very young children. In our opinion, this limitation applies when some pain exists, nevertheless at lower end of the spectrum (no pain status) pain is assessable in small children and permits rating. Nonetheless, the study’s strengths lie in its homogeneous, single-center population, consistent access to imaging and functional evaluation, and the use of objective, composite criteria to define optimal outcomes.

## Conclusions

In late-diagnosed DDH among children aged 1–4 years, a structured surgical protocol combining open reduction, femoral shortening with derotation and pelvic extracapsular osteotomy offers the highest likelihood of achieving optimal anatomical and functional outcomes. Achieving primary intraoperative stability facilitates shorter immobilization in a physiological position and may explain the low incidence of AVN observed. This approach may effectively restore joint congruency and delay or prevent degenerative changes in adulthood.

## Data Availability

No datasets were generated or analysed during the current study.
